# Evaluation of an individualized, tablet-based physiotherapy training programme for patients with Parkinson’s disease: the ParkProTrain study, a quasi-randomised controlled trial

**DOI:** 10.1186/s12883-022-02647-9

**Published:** 2022-05-14

**Authors:** Lynn Wagner, Björn Hauptmann, Ann-Kristin Hoffmann, Nicole Jochems, Bastian Schmeier, Andreas Schrader, Thomas Kohlmann, Ruth Deck

**Affiliations:** 1 Department Rehabilitation Sciences, Institute for Social Medicine and Epidemiology, University of Lübeck, Ratzeburger Allee 160, 23562 Lübeck, Germany; 2 Neurological Centre, Segeberger Kliniken GmbH, Bad Segeberg, Germany; 3 Department Performance Neuroscience, Therapy and Health, Medical School Hamburg, Hamburg, Germany; 4 Institute for Multimedia and Interactive Systems, University of Lübeck, Lübeck, Germany; 5 Institute of Telematics, University of Lübeck, Lübeck, Germany; 6 Methoden der Community Medicine, Universität, Greifswald, Germany

**Keywords:** Parkinson's disease, Quality of life, Participation, Exercise, Physiotherapy, Physical therapy, App, Tablet, Training programme

## Abstract

**Background:**

Regular physical activity is of great relevance in Parkinson's disease (PD). It is part of the inpatient multimodal Parkinson's complex treatment (MKP) in Germany. However, there is often a lack of human resources in outpatient settings to continue an interprofessional approach. A large proportion of PD patients live a predominantly sedentary lifestyle and do not get enough exercise.

**Methods:**

The intervention group (IG) used a tablet-based physiotherapy training programme at home for a period of nine months. We conducted a quasi-randomised longitudinal study with three measurement times (at the beginning (t_0_) and end of MKP (t_1_) and at 9 months after MKP (t_2_)). The primary outcome measured was PD-specific quality of life using the PDQ-8. The secondary outcome focused on participation restrictions, falling anxiety, sleep disorder, anxiety and depression as well as comorbidity, pain, performance capability and physical activity.

**Results:**

For *n* = 93 IG and *n* = 137 control group (CG) patients, evaluable cases were available for all measurement times. Both groups achieved significant improvements in all parameters at the end of MKP. These parameters deteriorated again at nine months after MKP for most parameters and were even below the baseline levels. However, this deterioration was less pronounced in the IG than in the CG. For general health and social participation, a significant slightly positive effect was observed in the IG nine months after MKP when compared with the baseline level. Paying attention to physical activity slightly increased in the IG for the catamnesis survey compared to baseline. Nearly all IG patients were satisfied with the intervention, especially with the consultations with the physiotherapist.

**Conclusions:**

Although the expected extent of effects could not be determined for the IG, stabilisation effects could be demonstrated. These stabilisation effects shown for the IG might be attributed to the intervention. The effects might have been greater without the COVID-19 pandemic.

Trial registration.

German Register of Clinical Trials, drks.de. Identifier: DRKS00014952. Registered 20/06/2018. Date and version identifier 25/04/2019; version 1.

## Background

Parkinson's disease (PD) is the second most common neurodegenerative disease after dementia [1]. Globally, the number of people that are affected will increase from 6.1 million people in 2016 to 9.3 million people in 2030 [2, 3]. The number of PD patients is also rising in Germany [4]. The disease brings immense costs to the German health care system [5]. For those affected, PD means great physical and psychological suffering [6]. The use of various forms of therapy represents an important component in the therapy for PD patients, in addition to the correct drug therapy [7]. A positive influence of physical activity on the quality of life of those affected has been demonstrated by numerous studies [8–11]. Physiotherapeutic interventions are part of the MKP, according to OPS 8-97d in Germany. The improvement of the general health and quality of life of those affected were focused on during a typical 14 to 21 day stay at the clinic [12]. Physiotherapy is recommended in the German S3 guidelines for “Idiopathic Parkinson’s Syndrome” as a flexible and long-term treatment strategy. In addition to the treatment itself, some physiotherapeutic interventions also focus on the motivations for physical activity [13]. This is important, as a large proportion of PD patients live predominantly sedentary lifestyles [14, 15]. There is often a lack of human resources in outpatient settings to continue an interprofessional approach to treat PD patients after MKP [12, 16]. It is very important to motivate people with Parkinson's disease to take responsibility for their own regular physical activity [13]. Digital applications have the potential to support patients with Parkinson's disease in the outpatient setting [17].

### Objectives

The ParkProTrain study aimed to evaluate the effectiveness of tablet-based physiotherapy compared to usual care on quality of life and other health-related parameters.

## Methods

A quasi-randomised, longitudinal effectiveness study (sequential study design) was conducted with three measurement times: at the beginning (t_0_) and end of MKP (t_1_) and 9 months after MKP (t_2_). The study has both qualitative and quantitative parts (a mixed–methods study). In this publication, the results of the quantitative part are reported. This methodological component is described in detail under section A in the study protocol, which has been published elsewhere [18].

### Sample size

To estimate the required sample size, we have referred to the results of existing studies on the influence of physical activity on quality of life (PDQ-8) in PD patients. For the effect determination, the data of Ebersbach et al. [19], Morris et al. [20] and Nadeau et al. [21] were being consulted. In Ebersbach et al., the quality of life over a 4-month course showed effect sizes (ES) of average magnitude (ES = 0.47) for patients who underwent physical training (Nordic walking), whereas there was no change in the CG. Morris et al. also report a significantly improved quality of life, with a mean effect size of ES = 0.45, after muscle training over a 3-month span. In their controlled study on the effect of intensive treadmill training (speed and incline) on quality of life, Nadeau et al. found an effect size of ES = 0.73 over a 6-month course; for simple treadmill training, they found an effect strength of ES = 0.21. In the CG, the quality of life did not change. For the calculation of the number of cases, we assume that participation in the tablet-based training programme (IG) will lead to clinically relevant positive effects (t_0_ vs. t_2_ ES = 0.40) on quality of life (PDQ-8) 9 months after the end of MKP. In contrast to the IG, there will be no changes in the CG at t_2_. To demonstrate differences between the IG and CG 9 months after MKP on the order of at least ES = 0.4 with two-sided testing at α = 5% and a power of 80%, a group size of *n* = 100 net was required for each IG and CG. We expected only a moderate dropout rate of 25% among patients after MKP due to their attachment to the clinic. Thus, to be able to evaluate *n* = 100 patients per group, 133 participants per study group were initially to be included.

### Study participants

The recruitment of CG and IG took place monocentrically at a specialist clinic for movement disorders in Germany (Fachklinik für Parkinson und Bewegungsstörungen, Segeberger Kliniken GmbH in Bad Segeberg). A physiotherapist^1^ at this clinic was in charge of screening the PD patients for their eligibility within their first three days of MKP and for recruiting them into the study.

Patients who were suffering from idiopathic Parkinson’s syndrome (IPS) and who were participating in a three-week MKP were included in the study. Patients with a MoCA (Montreal Cognitive Assessment) score [22] below 18 points and BBS (Berg Balance Scale) score [23, 24] below 41 were excluded from the study participation. Patients who were suffering from a major depressive episode, cardiovascular or orthopaedic/surgical or other health problems were also excluded. A diagnosis of moderate to severe dementia also led to exclusion. In addition, the patients were required to possess a sufficient knowledge of the German language to be able to both complete the questionnaires and perform the training with the German-language app. The study participants for the two groups were obtained from two recruitment phases, and only when the required number of patients for the CG was reached, the recruitment of the IG started. This sequential approach was chosen due to the different therapy regimens that made it impossible to manage both groups at the same time at the partner clinic. Neither the participants nor the study personnel or care providers at the clinic were blinded.

### Study outcomes

The participants in the CG and IG completed written questionnaires using standardised, validated instruments. In addition, the patients in the IG were asked intervention-specific questions. Table 1 shows the core set of instruments used. A detailed description of the instruments is provided in the study protocol [18].

**Table 1:** Core set of instruments

Dimensions	Instruments	t_0_	t_1_	t_2_
**Primary Outcome**				
Quality of Life	PDQ-8 [25]	•	•	•
**Secondary Outcomes**				
Participation Restrictions	IMET [26]	•		•
Fear of Falling	FES-I [27]	•	•	•
Sleep Disorder	PDSS-2 [28]	•	•	•
Anxiety / Depression	PHQ-4 [29]	•	•	•
Comorbidity	SCQ-D [30]	•		•
Pain	Single Items [31]	•	•	•
Performance Capability	Single Items [32, 33]	•		•
Physical Activity	Federal Health Survey [34]	•		•
**Moderating Variables**				
Body Height, Weight	Single Items	•		•
Use of Health Services	Single Items	•		•
Sociodemographic Data	Single Items [35]	•		•

t_**0**_ = baseline/right before MKP; t_**1**_ = 3-week follow-up/right after MKP; t_**2**_ = 9 months after t_**1**_

### Intervention programme

As part of ParkProTrain, a physiotherapy training programme was developed as a tablet-based app. It was designed to help PD patients to continue physical activity in their daily lives after discharge from the clinic. The app contains videos with verbal instructions and explanations for all the physically activation exercises taught in the MKP. These exercises are available in different degrees of difficulty and promote endurance, strength and balance. The programme makes it possible to compile individual training plans from the exercises. This was done by the clinic's physiotherapist for the IG patients. The training plans were regularly adapted to the needs of the patients. If a training session was performed by a patient, this was automatically saved in the app's calendar. Furthermore, patients can add additional endurance sessions to the app. The detailed design of the video-based training programme has been published elsewhere [36].

Within the study the CG received the usual MKP therapy services and, after discharge, the usual outpatient treatment. The IG also underwent MKP but was introduced to the previously developed tablet-based training programme in the process. In the introductory stage, the physiotherapist conducted three patient-centred seminars. In addition, initial individualized training plans were developed in close consultation between the physiotherapist and patient. The training programme could be used during the hospital stay. The actual nine-month intervention started after discharge from the clinic. Patients were asked to train using the programme for up to three times a week, in addition to their usual outpatient therapy. Patients were also asked to complete an endurance workout once a week and to enter it in the app. A personal exchange between the patient and physiotherapist took place every three weeks. Originally, a combination of face-to-face and telephone meetings was planned. Due to the contact restrictions during the COVID-19 pandemic, the meetings were held by telephone. The exchange was mainly used to assist the study participants in conducting the training sessions in their homes and in staying motivated. For the physiotherapist, the exchange was helpful for creating updated individualized training plans every nine weeks. In addition to the training programme, the IG patients took part in the usual outpatient therapy, just like the CG.

**Fig. 1:**
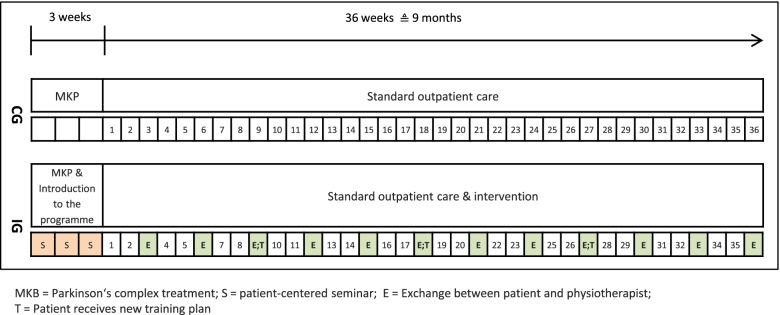
Organisational flow of the study

Figure 1 illustrates the organisational flow of the study.

### Data analysis

Mean values, standard deviations and absolute/relative frequencies were used as descriptive statistics for continuous and categorical data, respectively. Statistical significance of differences between groups or between time points was assessed using t-tests and Chi-squared tests (Pearson or McNemar tests, as appropriate). To address the main effectiveness research question, we conducted analysis of variance for repeated measures. Time, group and time by group interaction effects are reported. Only complete cases were used in the statistical analyses. The analyses were performed using the statistics programme SPSS 22.0. In addition, we calculated the standardised response means (SRM) to describe changes in continuous variables over time. SRMs are calculated by expressing the absolute mean differences in terms of the standard deviation of the differences [37]. These were interpreted according to Cohen: d > 0.2 small effect, d > 0.5 medium effect, and d > 0.8 large effect [38]. The significance level was set to *p* < 0.05.

## Results

### Demographic sample characteristics

**Fig. 2:**
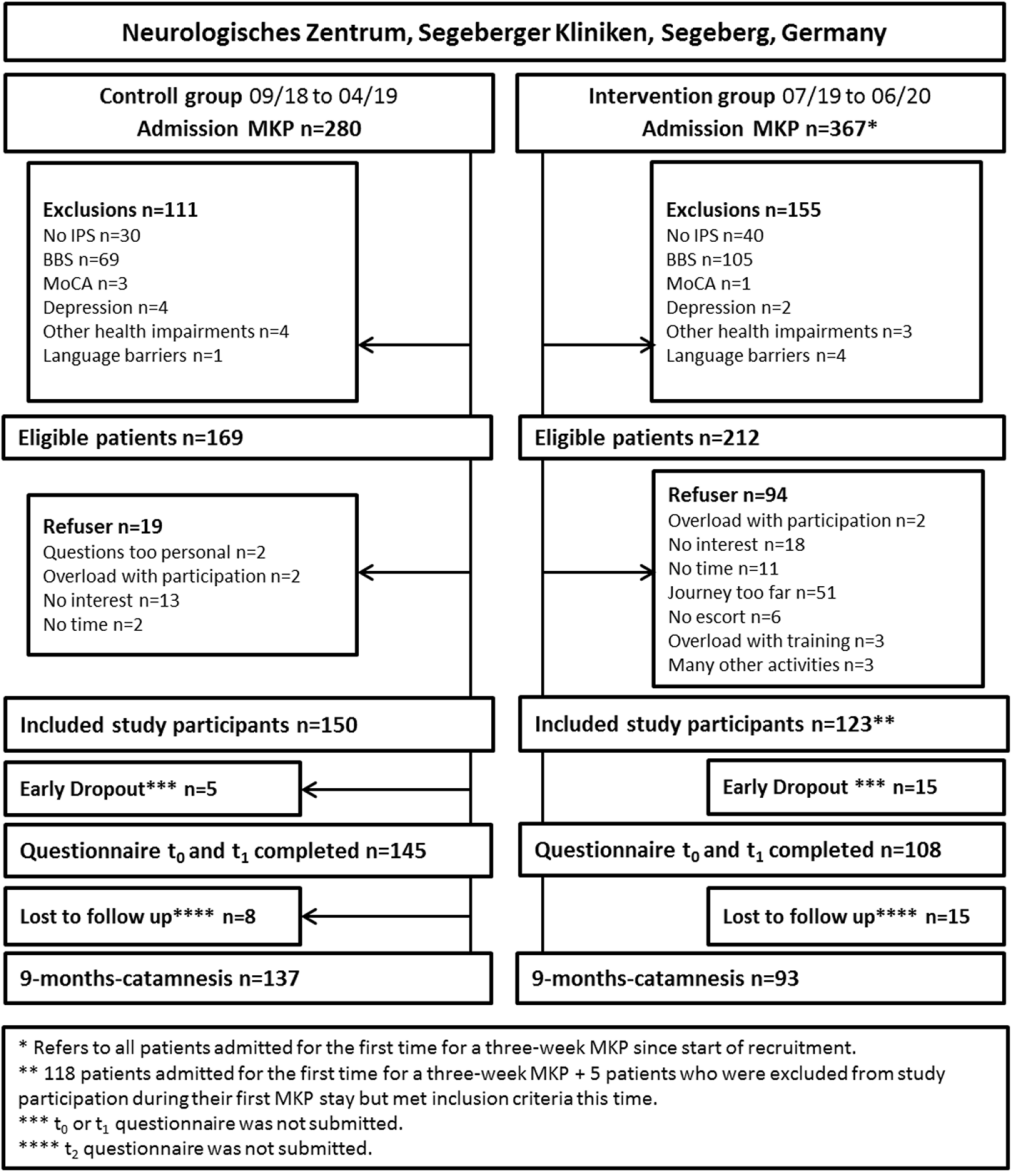
Sample flow

Recruitment took place from September 2018 to June 2020. After the assessment and review of the inclusion and exclusion criteria, *n* = 386 patients were asked to participate. *N* = 113 of these eligible patients refused to participate. A total of *n* = 273 PD patients were willing to participate after being informed about the study and data protection and included *n* = 123 IG and *n* = 150 CG participants. For a total of 230 PD patients, the data for all three measurement times were available, which included *n* = 93 for IG and *n* = 137 for CG. Follow-up data collection took place from June 2019 to January 2020 for the CG and from April 2020 to April 2021 for the IG. The detailed sample flow is shown in Fig. 2.

The demographic and Parkinson-related characteristics of the IG and CG are listed in Table 2. On average, the patients had been suffering from PD-specific symptoms for approximately 9 years. The disease was diagnosed at an average of approximately 8 years prior to the study. The sample groups included more men than women in both groups. There were significant differences in age, education, household net income and occupational status, BMI, and degree of disability. The average age for the IG was 64 years, and for the CG, it was just under 68 years. The participants in the IG had, on average, higher educational levels. In the IG, just under 30% and in the CG, 16% were employed. The disease severity, according to Hoehn & Yahr, averaged approximately 2.5 for both groups.

**Table 2:** Sample characteristics

	IG *(n* = *93)*	CG *(n* = *137)*	*p*-value^a^
Mean age, years (SD)	64.1 (9.3)	67.6 (9.3)	** < 0.01**
Gender, n (%)			0.427
*Male*	62 (67.4%)	84 (62.2%)	
*Female*	30 (32.6%)	51 (37.8%)	
School education n (%)			**< 0.01**
*Max. main school*	18 (19.8%)	60 (43.8%)	
*Secondary/Polytechnic school*	34 (37.4%)	39 (28.4%)	
*High school diploma*	39 (42.9%)	38 (27.7%)	
Marital status, n (%)			0.649
*Married*	66 (74.2%)	104 (76.5%)	
*Single*^*1*^	23 (25.8%)	32 (23.5%)	
Household net income, n (%)			**0.039**
*Low (under € 1500)*	10 (12.2%)	19 (15.6%)	
*Medium (€ 1500 to under € 3000)*	39 (47.6%)	68 (55.7%)	
*High (€ 3000 or more)*	33 (40.2%)	35 (28.7%)	
Occupational status, n (%)			
*Currently employed*	26 (28.3%)	21 (15.8%)	**0.024**
Parkinson’s disease, M (SD)			
*Years since diagnosis*	7.75 (6.2)	8.23 (5.1)	0.525
*Years since symptoms*	8.89 (6.3)	9.11 (5.6)	0.778
*Disease severity (Hoehn&Yahr)*	2.57 (0.7)	2.54 (0.7)	0.707
Body Mass Index, M (SD)	28.1 (5.2)	26.5 (5)	**0.021**
Disability			
*Recognised disability, n (%)*	69 (75.0%)	103 (76.3%)	0.824
*Degree of disability, M (SD)*	50.6 (15.3)	62.7 (18.4)	**0.041**

^a^Chi^2^/ T-Test; ^1^hereunder single, widowed, divorced, separated living

*M* Mean Value, *SD* Standard Deviation, *n* number

### Loss to follow-up

To assess the risk of bias due to dropouts, a nonresponder analysis was conducted for the IG and CG. The sociodemographic, as well as primary and secondary outcome measures, were examined. For both the IG and CG, there were no significant differences with regard to the examined outcomes.

#### Primary and secondary outcomes

Most of the primary and secondary outcomes were comparable at baseline in both groups. The values indicated the burdens in both the IG and CG. Participation was statistically significantly more limited at the start of MKP in the CG than in the IG. For the PHQ-4 total scores, the IG also achieved significantly better values than the CG. With regard to performance, it was noticeable that those who were employed (IG: *n* = 26, CG: *n* = 21) felt very limited in this area. In everyday life, a greater number of CG patients felt that their performance was significantly inferior compared to the IG patients.

Both the IG and CG achieved significant improvements at the end of MKP with small to medium effect sizes for all target parameters. Both groups benefited from MKP to a similar extent. There were only significant differences in the group comparisons for the FES-I, PHQ-4 total score and PHQ depressiveness variables. There were no significant differences between the groups over time. There also were no significant differences with regard to pain at the end of MKP.

For both the CG and IG, a decrease in the effect on the primary outcome (quality of life) at nine months after MKP, when compared to the baseline, was recognisable but was less pronounced for the IG than CG. The difference did not reach statistical significance. The patients in both groups deteriorated in terms of nearly all primary and secondary outcome variables at catamnesis. However, this deterioration was less pronounced in the IG than in the CG. For the IMET, a significant slightly positive effect was observed in the IG. The IG remained slightly below baseline. The primary and secondary outcomes over time are shown in Table 3. There were no significant differences between the groups in terms of medically diagnosed comorbidity and pain at nine months after the end of MKP.

**Table 3:**
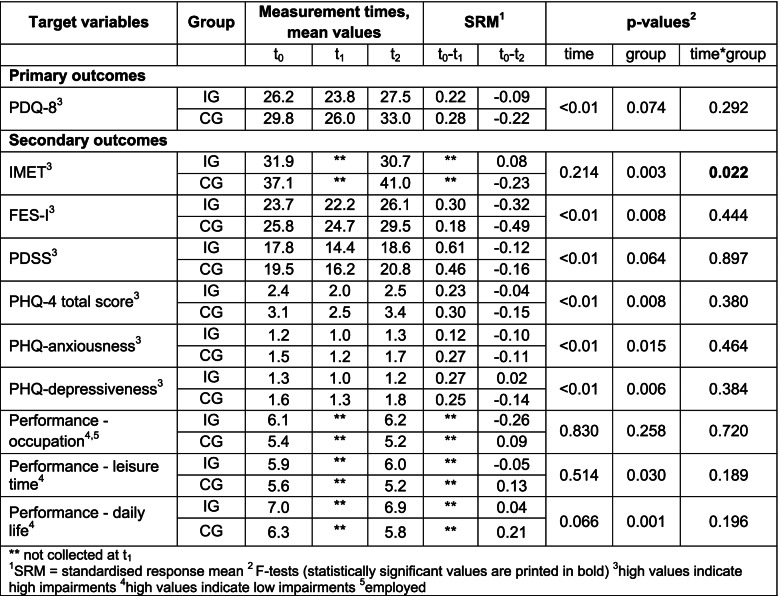
Primary and secondary outcomes over time

#### Health status

The general health status of the patients in the IG and CG at t_0_ was similarly distributed. Only approximately 18.5% of IG and 13.2% of CG participants described their health status as “very good” or “good”, whereas approximately 34.7% of IG and 46.3% of CG perceived their health status as “less good” or “poor”. Approximately one-fifth of the IG patients (*n* = 19) had fallen an average of 8.5 times (SD = 17.1) in the 6 months before MKP. In the CG, almost half of the patients reported having fallen (*n* = 57) and had fallen an average of 6.2 times (SD = 19.8). In addition to PD, the study participants had an average of two other diseases. High blood pressure was reported as the most common comorbidity by both the IG and CG, which was followed by osteoarthritis and elevated blood lipid levels. The patients in both groups predominantly reported pain in the back and shoulder–neck area and in the lower limbs.

The general health of the participants improved in both groups at the end of MKP. Forty-four percent of the IG and approximately one-third of the CG patients (32.6%) described their own state of health as "good" or "very good". Nearly 40% of the IG and 45% of the CG respondents perceived their state of health to be "satisfactory". There were no statistically significant differences between the groups. The improvements in health status compared to that before the MKP were statistically significant, with an SRM of 0.51 for the IG and 0.55 for the CG (*p* < 0.01).

With regard to the number of falls during MKP, the groups did not differ: *n* = 4 IG patients fell an average of 2 times, and *n* = 17 CG patients fell 2.2 times.

**Fig. 3:**
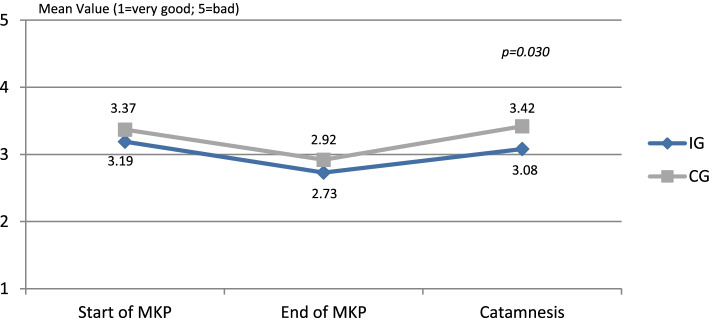
Health status over time

Nine months after MKP, the CG patients rated their general state of health as significantly worse than the IG patients (Fig. 3). Thus, 29.2% of the IG and approximately half of the CG respondents (50.4%) assessed that their state of health was "less good" or "poor". When examining their health status over time, it was noticeable that it improved slightly in both groups after MKP. Nine months after the end of MKP, however, it deteriorated again. For the case of the IG, however, the state of health did not decrease to the initial level but remained slightly better. For the case of the CG, on the other hand, the state of health deteriorated beyond the initial level (SRM: CG (t_0_-t_2_): -0.07, IG (t_0_-t_2_): 0.12).

On average, *n* = 39 IG patients fell 5 times and *n* = 59 CG patients fell almost 8 times in the first nine months after MKP. The difference between the groups did not reach statistical significance.

#### Use of health services

Nearly all patients in both groups at baseline had sought medical help in the previous six months. Eighty-eight percent of the IG and 85% of the CG patients had seen a neurologist in the previous six months before the start of MKP, and nearly 82% of the IG and 84% of the CG patients had seen a general practitioner. A total of 80% of the patients in both groups had used the services of one or more therapists in the previous six months. The patients in both groups were most often in physiotherapeutic treatment, and this was statistically significantly more often for the CG than the IG. *N* = 28 patients with IG (36.4%) and *n* = 49 patients with CG (63.6%) had been hospitalised in the previous 6 months before MKP onset. In addition to Parkinson's-specific medications, few others were taken. Antidepressants/psychotropic drugs were taken daily by 19% of the IG and 16.1% of CG patients. Ten percent of the IG and 15.7% of CG patients took sedatives and sleeping pills daily. More than half of the patients (IG: 54.1%, CG: 59.5%) also took other prescription drugs daily.

#### Physical activity

The IG and CG did not differ significantly in terms of physical activity at baseline. Approximately one-third of the IG (33.3%) and CG patients (38%) were physically active for more than two hours a week at the start of MKP. Likewise, approximately one-third of the study participants (IG: 34.4%; CG: 32.1%) were active 1–2 times a week. Another third (IG: 32.3%; 29.9%) exercised less than once a week. A total of 44.1% of the IG patients and 51.1% of the CG patients stated that they paid either much or very much attention to engaging in adequate amounts of physical activity. None of the patients in either the CG or IG stated that they paid no attention to physical activity.

In both groups, the proportions of patients who were physically active for more than two hours a week increased nine months after MKP. The groups did not differ significantly from each other at either measurement time, and no significant effects were detected over time (Table 4).

**Table 4:**
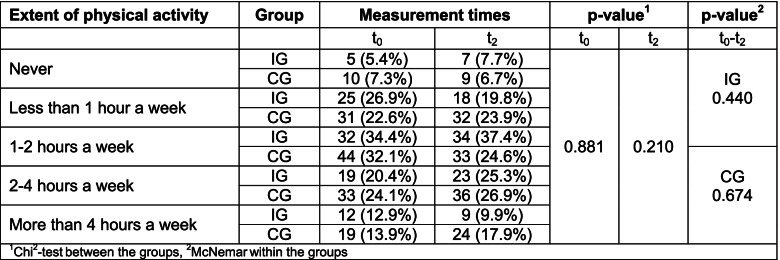
Extent of physical activity over time

The proportions of patients who paid much to very much attention to exercise increased slightly in the IG group in the catamnesis survey, while the proportions decreased slightly in the CG. There were no significant differences at the group level or over time (Table 5).

**Table 5:**
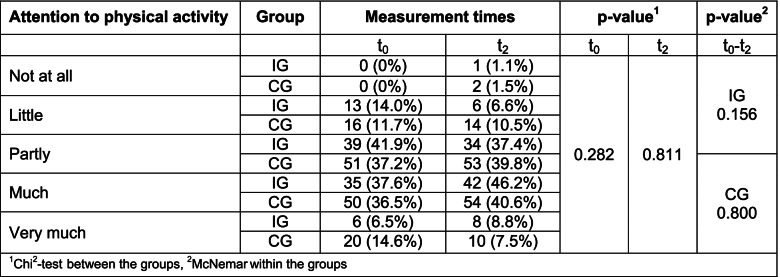
Attention to physical activity over time

#### Occupational participation

At statistically significant levels, more patients in the IG were employed at baseline than in the CG (IG: *n* = 26, 28.3%; CG: *n* = 21, 15.8%). In the previous six months, *n* = 22 employed IG patients had been on sick leave for an average of nearly three weeks, and *n* = 19 CG patients had been on sick leave for an average of nearly 2.5 weeks. These differences in duration did not reach statistical significance. Occupational risks were calculated using the SPE scale (a scale for measuring the subjective prognosis of employment) [39, 40]. In both the IG and CG, the risk scores were high in slightly more than half of the patients. More than half of the IG patients and nearly two-thirds of the CG patients who were still working assumed that they would not be able to work until they reached retirement age. Nearly 70% of the IG and nearly 80% of the CG patients believed that their ability to work was permanently at risk. Approximately 40% of the patients in both groups were currently considering applying for a disability or occupational disability pension. There were no statistically significant differences between the groups for any of the described variables. *N* = 14 of the employed IG, and *n* = 12 of the employed CG participants stated that their professional situations had changed in the previous three years because of their PD.

Additionally, nine months after MKP, significantly more patients in the IG (*n* = 23, 25%) than in the CG (*n* = 15, 11.8%) were employed. Thus, there was one fewer employed patient in the IG and six fewer employed patients in the CG than at baseline. The sick leaves in the previous nine months lasted approximately three weeks in the IG and CG. For the IG, the number of sick leaves thus remained more or less the same, while the patients in the CG were on sick leave for half a week longer on average than at baseline. The difference is not significant. Approximately half of the patients in both groups belonged to the two highest risk groups, which approximately corresponded to the baseline value.

#### Intervention

**Fig. 4:**
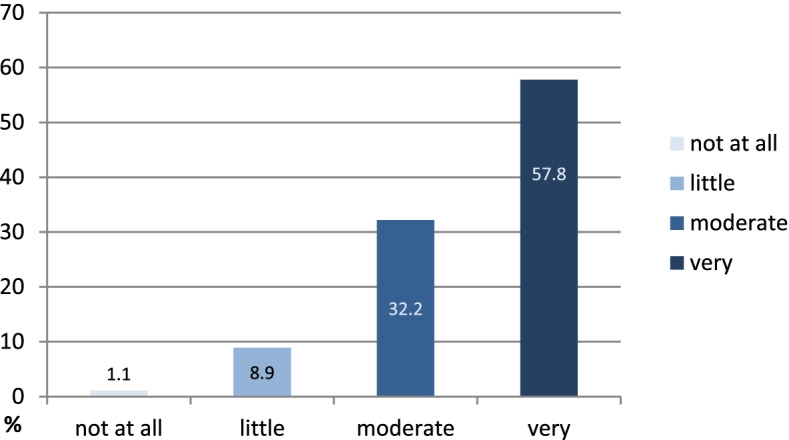
Assessment of motivation for physical activity through the training programme

Ninety percent of all IG patients felt “moderately motivated” or “very motivated” by the training programme to become physically active (Fig. 4).

**Fig. 5:**
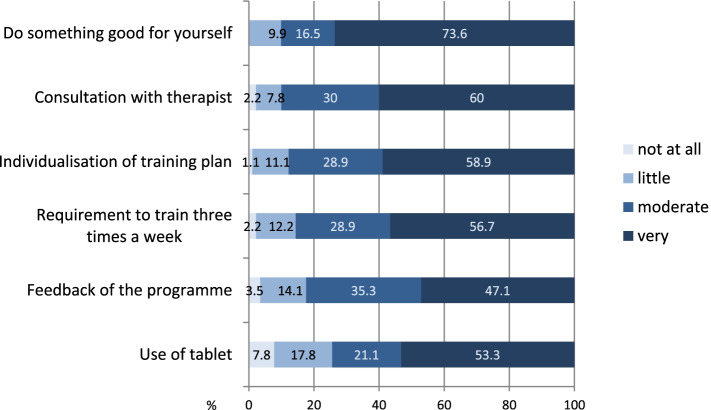
Assessing the motivation of individual aspects of the training programme

Individual aspects of the intervention motivated the patients to exercise. The most motivating aspects for the IG were the idea of doing something good for their own bodies by performing the exercises and having regular consultations with the physiotherapist, which were followed by the continuous adjustments of the training plan and the requirement to train three times a week (Fig. 5).

The patients found that both regular telephone calls with the physiotherapist (“very helpful” and “moderately helpful”: 85.6%) and introductory seminars in the clinic (“very helpful” and “moderately helpful”: 75.6%) were mostly helpful to enable them to use the training programme at home.

**Fig. 6:**
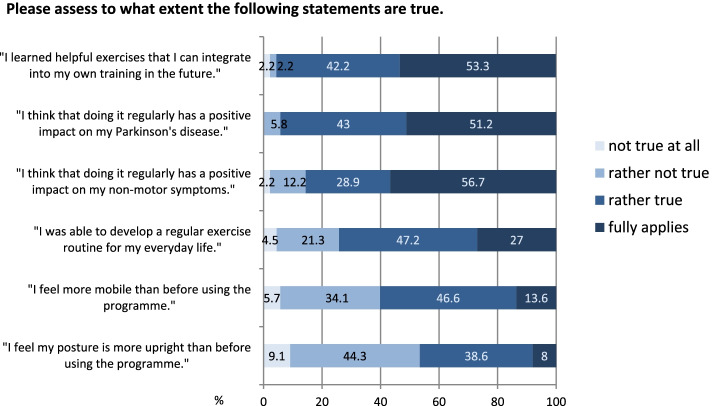
Assessment of individual statements on the training programme

All patients benefited from using the programme ("somewhat" to "a lot": 94%). Above all, the patients benefited from the fact that they learned helpful exercises for use in their future self-training. The majority of patients also felt that regular performance of the exercises had a positive influence on their PD (Fig. 6).

**Fig. 7:**
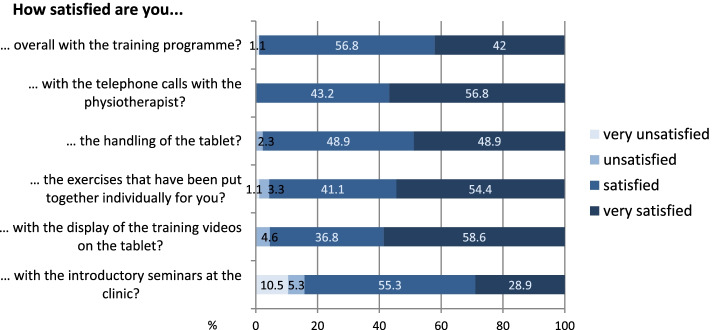
Assessment of satisfaction with individual aspects of the training programme

Overall, the patients were satisfied with the training programme (“satisfied” to “very satisfied”: 98.8%). The aspects that contributed most to this satisfaction were telephone calls with the physiotherapist, using the tablet, the individually focused training plans and the training video presentations (Fig. 7).

Only 16.7% of the IG patients reported operating difficulties with the programme, which occurred between one and eight times. Some technical problems occurred during the intervention period. The most frequently mentioned issues were intermittent sound drop outs, a programme crash and inadequate programme volume.

#### Statistical control of the differences in baseline values of the outcome variables and sociodemographic characteristics

A regression analysis was conducted to control for the differences in primary and secondary outcomes (baseline values) as well as for the sociodemographic characteristics (age, education and occupational status) at baseline. The analysis showed that the previous results for the primary outcome, PDQ-8, and for the secondary outcome, IMET, were mostly replicated.

In the analysis of the other secondary outcomes, statistically significant differences in the change values that favoured the IG were found for the total PHQ-4 scores, PHQ-depression scores, performance (leisure time as well as performance) in daily life (when controlling for the differences in baseline values) and for the PHQ-depression scores, performance—leisure time as well as performance—daily life (when controlling for the differences in baseline values and socio-demographic characteristics) at the second follow-up time point.

## Discussion

Both groups benefitted from the MKP measures. The health statuses as well as the primary target variables for quality of life and all secondary target variables improved with small to medium effect sizes in both the IG and CG at the end of MKP. The health statuses of both groups deteriorated nine months after MKP when compared to the end of MKP. In the CG, the parameters even worsened beyond their baseline levels. For the IG, the baseline levels were more or less maintained when examining the primary and secondary outcomes. For participation and depression, the IG values were even slightly better than those at the beginning of MKP. When controlling for the differences in baseline values as well as the sample characteristics, the other secondary target parameters for the IG improved.

According to the results of the interviews conducted in weeks 9 and 36, the patients were motivated to use the training programme. The majority of the patients trained for the entire nine-month intervention period. For some, only illness led to training interruptions in the meantime [41]. The adherence of the patients was also confirmed by the evaluation of the training data.

Nevertheless, with regard to physical activity, there were no increases in frequency for the IG. Only a few more IG patients paid attention to their physical activity, but this effect was not significant over time or between the groups. Training with the programme was originally intended to be carried out by the patients in addition to standard outpatient physiotherapy and other activities. If these activities had been performed to approximately the same extent as before the MKP, the physical activity should have increased many times over. The following can explain why the expected values were not met: All IG patients, in contrast to the CG patients, were affected by the limitations associated with the COVID-19 pandemic during catamnesis. The interviews that were conducted with patients within this study showed that during the COVID-19 lockdown in spring 2020, temporary outpatient physiotherapy was not conducted. Additionally, sports facilities, such as gyms and swimming pools, were closed for long periods. First, sources indicated limited physical activity during the pandemic. This lack of activity concerned, among others, people who are in the second half of life [42], and the patients in our sample are predominantly of this age. The initial data are also available for obese patients with increased inactivity-associated health risks and show reductions in the frequency physical activity [43]. In a study [44] that surveyed PD patients who were predominantly from the USA, just under half of the patients (44.7%, *n* = 600) reported that they reduced their physical activity during the pandemic. A large proportion (72.9%, *n* = 978) also reduced their activities outside the home, including participation in fitness classes and nonprimary sports activities. To what extent these Parkinson's-specific results can be applied to the German population is not clear due to differences in the COVID-19 related circumstances and their associated measures. However, it can also be assumed that German PD patients with existing inactive lifestyles [15, 45] further limited their exercise during the pandemic. In pandemic situations but also in other contexts, the tablet-based training programme can complement or even temporarily replace physiotherapy. This was also shown by other studies [46]. If implemented in everyday care, the training programme might lead to cost reductions when compared to the complementary use of conventional physiotherapy. There is a need for further research in this area.

Furthermore, the study showed that the training programme was carried out independently and regularly. The majority of patients felt motivated to become physically active. Past studies showed that motivation of PD patients can be achieved by addressing specific personal barriers and motivators, e.g. by a physiotherapist. This was implemented in ParkProTrain and could also be recommended for future studies [47].

Overall, the patients were satisfied with the intervention. Above all, the introductory seminars given at the clinic as well as the regular contact with the physiotherapist and the adaptation of the training programme to individual needs during the intervention were indispensable components of the intervention in addition to the technical components of the programme. Another study with a similar multifaceted approach combining an app-based training in the home with supervision also showed good adherence [48].

### Limitations

Some significant differences in the sample characteristics of the CG and IG can be found. At the same time, it was noticeable that the trips to planned face-to-face meetings were the main reason for study denials. It can be assumed that inactive patients did not want to manage the planned journeys to the clinic. We might have therefore unintentionally excluded this group of patients from study participation. Due to the COVID-19 related circumstances, these face-to-face meetings at the clinic could not be taking place. We learned from our study that telephone calls are sufficient for exchanges with the physiotherapist. For future projects, we would therefore recommend planning only telephone contacts/video consultations or at least, reducing the number of personal meetings to a minimum.

## Conclusions

We can positively conclude that despite the impacts of the COVID-19 pandemic on the training programme, the stabilisation effects of MKP can still be detected in the IG sample. These were not measurable in the CG, which was not affected by the pandemic during the data collection period. We assume that if the intervention had been implemented under nonpandemic circumstances, the effects would have been much more positive. At the same time, the pandemic and the accompanying contact restrictions make the development and implementation of these kind of remote interventions more urgent. Overall, we must conclude that the expected health-related results, such as the positive influence on the quality of life, could not be proven. The individual components of the ParkProTrain intervention seem to have been well chosen. In particular, the close contact between physiotherapist and patients during the MKP (introductory seminars) and during the entire intervention (regular telephone contacts) seems to have been helpful for good adherence.

## Data Availability

The datasets generated and/or analysed during the current study are not publicly available. Declaration of consent does not include release of the data for external analyses. A model consent form is available from the corresponding author by reasonable request. Trial results will be published and thus be made available to anyone interested in the study.

## Abbreviations

App: Application
BBS: Berg Balance Scale
CG: Control group
IG: Intervention group
IPS: Idiopathic Parkinson’s syndrome
IMET: Index zur Messung von Einschränkungen der Teilhabe (Measurement of Restrictions on Participation)
ISE: Institute for Social Medicine and Epidemiology
MKP: Multimodale Komplexbehandlung bei Morbus Parkinson is a mostly inpatient three-week programme for PD patients in which they receive drug therapy as well as units in physiotherapy, occupational, music, sports and speech therapy and neuropsychology
MoCA: Montreal Cognitive Assessment
n: number
OPS: Operationen- und Prozedurenschlüssel (the German Operations and Procedures Key) is used to encode the operations and medical procedures for inpatient care as well as for outpatient surgery in the clinic
p: probability
PD: Parkinson’s disease
SD: standard deviation
SPSS: Statistical Package for the Social Sciences
SPE: scale for measuring the subjective prognosis of employment
SRM: standardised response mean

## Footnotes

1 In the following, "physiotherapist" refers to the Neurological Centre physiotherapist who supervised the recruitment as well as study implementation.
